# Assessment of skeletal maturity using the calcification stages of permanent mandibular teeth

**DOI:** 10.1590/2177-6709.23.4.44.e1-8.onl

**Published:** 2018

**Authors:** Adeel Tahir Kamal, Attiya Shaikh, Mubassar Fida

**Affiliations:** 1 The Aga Khan University Hospital, Department of Surgery, Section of Dentistry (Karachi, Pakistan). Aga Khan University The Aga Khan University Hospital Department of Surgery Section of Dentistry Karachi Pakistan; 2 Liaquat College of Medicine and Dentistry, Dar-ul Sehat Hospital (Karachi, Pakistan). Liaquat College of Medicine and Dentistry Dar-ul Sehat Hospital Karachi Pakistan

**Keywords:** Cervical vertebrae, Maturity, Age

## Abstract

**Introduction:**

Knowledge of the growth status of patients is essential to formulate and initiate a precise treatment plan. This study aimed at determining the role of calcification of permanent mandibular teeth for the assessment of skeletal maturity.

**Methods:**

A cross-sectional study was conducted using lateral cephalograms and dental panoramic radiographs of 360 patients (ages 7-18 years) equally divided into six groups according to cervical vertebral maturation stages. Skeletal age was determined using Baccetti et al. method and dental age was calculated using Nolla and Demirjian methods.

**Results:**

Mean chronological stage at CS5 revealed a significant difference between male and female subjects (*p*= 0.003), which showed that the latter achieved skeletal maturity one year earlier than the former. A significant difference (*p*= 0.007) was found for dental age using Nolla’s stages at CS3, which showed females demonstrated a dental age of 1.4 years less than males. Mandibular canine showed the highest correlation with Demirjian index (DI) in males (rho = 0.818) and females (rho = 0.833). Mandibular second premolar showed the highest correlation with Nolla’s stages in males (rho = 0.654) and females (rho = 0.664).

**Conclusion:**

Comparisons between sexes revealed that females are skeletally and dentally advanced. The DI indicated stage F and Nolla’s stages identified stages 9, 10 to be indicative of CS2-3 for the mandibular canine and stages F and G and 9-10 for CS2-3 for the first premolars, second premolars and second molars, respectively.

## Resumo

**Introdução:**

o conhecimento acerca do *status* de crescimento dos pacientes é essencial para se formular e iniciar um plano de tratamento preciso. Esse estudo teve como meta determinar a correlação entre a calcificação dos dentes inferiores permanentes e a avaliação da maturação esquelética.

**Métodos:**

um estudo transversal foi conduzido utilizando-se radiografias laterais e panorâmicas das arcadas de 360 pacientes (idades entre 7 e 18 anos), igualmente divididos em seis grupos, de acordo com os estágios de maturação esquelética das vértebras cervicais. A idade esquelética foi determinada utilizando-se o método de Baccetti, e a idade dentária foi calculada utilizando-se os métodos de Nolla e Demirjian.

**Resultados:**

o estágio cronológico médio em CS5 revelou uma diferença significativa entre os indivíduos do sexo masculino e do feminino (*p*= 0,003), demonstrando que as meninas alcançavam a maturação esquelética um ano antes dos meninos. Encontrou-se uma diferença significativa (*p*= 0,007) para a idade dentária utilizando-se os estágios de Nolla em CS3, o que revelou que as meninas exibiam uma idade dentária 1,4 anos inferior à dos meninos. Os caninos inferiores demonstraram a maior correlação com o índice de Demirjian (DI) tanto em indivíduos do sexo masculino (rho = 0,818) quanto do feminino (rho = 0,833). Já os segundos pré-molares inferiores revelaram a maior correlação com os estágios de Nolla, tanto em meninos (rho = 0,654) quanto em meninas (rho = 0,664).

**Conclusão:**

as comparações entre os sexos revelaram que as mulheres são mais precoces tanto no desenvolvimento dentário quanto no esquelético. O DI indicou o estágio F, e o método de Nolla identificou os estágios 9 e 10 como indicativos de CS2-3, para os caninos inferiores; e os estágios F e G, e 9 e 10 para CS2-3, para os primeiros e segundos pré-molares, e segundos molares, respectivamente.

## INTRODUCTION

Age determination has great importance when treating growing orthodontic patients;^1^ therefore it is necessary that the clinician ascertain the growth status of the individual so that the appropriate treatment can be timely initiated.^1^ Orthodontic appliances might be prescribed to take advantage of growth periods to either overcome deficient skeletal growth or excessive growth.^2^ The inaccuracy in determining the patients’ growth status using the chronological age (CA) has been well established in literature.^3^^-^^5^ For this reason, Hassel and Farman^6^ developed a method through which the growth potential of an individual can be identified by the morphology of the cervical vertebrae. It was later modified by Baccetti et al,^7^ who used the lateral cephalogram to determine six growth stages based on the morphology and the presence of an indentation in the inferior border of cervical vertebrae. 

Differences between chronological and biological ages have led to the development of different indicators of maturity such as skeletal age (SA), morphological, sexual and dental ages (DA).^2^ DA can be assessed by observing the degree of calcification of the teeth on radiographs. Tooth calcification is a more reliable indicator of dental maturity than tooth eruption because it is not affected by local factors, such as loss of primary teeth, lack of space, malnutrition, dental decay, ankylosis, orthodontic anomalies, in addition to be under genetic control.^8-10,^

A number of classification methods have been developed to assess the DA.^8^^,^^9^^,^^10^ The most widely used dental maturity scoring system is the method developed by Demirjian et al^8^ in 1973 on a sample of French-Canadian children.^11^^-^^13^ The Demirjian index (DI) is frequently used to estimate dental age due to its simplicity, intra-examiner agreement, ease of standardization and ability to be reproduced. It has been used and tested across a wide range of populations.^11^^-^^13^ The Nolla’s stages^9^ (NS) were introduced in 1960 and a number of studies have found this method to be very accurate in different populations.^14^^-^^17^

The pubertal growth period influences diagnosis, treatment planning and the outcome of the treatment.^2^ It might be identified using cervical vertebral maturation method.^5^ Due to ethical concerns, not for every patient, a lateral cephalogram can be requested for the sole purpose of determining skeletal age. Instead, a dental panoramic radiograph can be used, which has a greater application in the diagnostic process.^18^ A relationship established between permanent mandibular teeth calcification stages and cervical vertebral maturation method may help in determining the skeletal maturity of the patient on the dental panoramic radiograph. Studies have been performed with a similar approach, however they have not have been able to assess dental maturation of all seven mandibular teeth and compile the results.^1^^,^^3^^,^^7^^,^^12^^,^^13^ Furthermore, there is a constant change in growth trends, variations in ethnicity and other reasons to reinvestigate this matter.^2^ Therefore, the objectives of this study were to compare male and female subjects’ chronological and dental ages in different skeletal stages, determine the correlation between dental and chronological ages, compare the percentage distribution of the calcification stages of each tooth among the growth peak and to determine the correlation of each tooth stage with skeletal age.

## MATERIAL AND METHODS 

A cross-sectional study was conducted using pretreatment dental panoramic radiographs and lateral cephalograms of patients presenting for orthodontic treatment in authors’ clinics after being approved by the Faisalabad Medical University’s ethical review committee (4117-Sur-ERC-16). The sample size was calculated using the findings of Baba et al,^19^ who found a correlation of 0.403 between cervical stages (CS) and mandibular canine in females. Keeping the power of the study as 90% and alpha as 0.05, the sample was calculated as 52. Since sex is a confounding factor^6^, equal number of subjects from both sexes was collected. In order to increase the power of our study, a maximum number of available subjects were included. The total sample of 360 [Male (n=180), Female (n=180)] subjects was further divided into six cervical vertebral stages (n=60) with equal number of males and females. The mean age of the patients was 12.5 ± 2.1 years (males: 12.8 ± 2.0, females: 12.2 ± 2.1). 

All subjects of Pakistani origin between 7-18 years of age who had good quality radiographs were consecutively selected and included in this study. Those subjects who had poor radiographic records, extracted or missing teeth, craniofacial syndromes, history of trauma or surgery involving facial structures and known cases of systemic diseases that affect the growth and development i.e. cleidocranial dysplasia, Down syndrome, cleft lip/palate, hypo and hyperparathyroidism and hypo and hyperthyroidism were excluded. 

All dental panoramic radiographs and cephalograms were standardized and were taken using Orthoralix R9200 (Gendex-KaVo, Milan, Italy). Cephalograms were taken with rigid head fixation and a 165 cm film to tube distance. The cervical vertebral morphology and developmental stages of the teeth were analyzed using the digital images of the radiographs on Rogan Delft View Pro-X (Rogan Delft B.V., Veenendaal, Netherlands) software.

The skeletal age was established on the lateral cephalograms using the Baccetti’s modified cervical vertebral maturation method.^7^

The dental age was determined using the dental panoramic radiographs of patients. Each of the left seven mandibular teeth, which included the central incisor, lateral incisor, canine, first premolar and second premolar, was staged according to the DI and NS. In order to estimate dental age using the Demirjian method,^8^ each tooth is given a mark indicating a developmental stage (ranging from A to H; there is also an additional stage 0, meaning no signs of mineralization).^8^ The sum of the obtained values indicated the dental age of the patient, which is derived from standard tables. Nolla^6^ has developed 10 stages of tooth mineralization and each tooth is staged accordingly. The score for each individual tooth is then summed to obtain a dental maturity score, which corresponds to the dental age. In order to study a direct relationship between the cervical stages and the seven stages of Demirjian’s method (B, C, D, E, F, G, and H), the percentage distribution of dental development stages in subsequent cervical stages was calculated taking sex into account. 

To test the intraexaminer reliability, 30 dental panoramic radiographs were randomly selected and the Demirjian dental maturity score and dental age and the NS and dental age were reevaluated by the principal investigator. The Intraclass Correlation Coefficient (ICC) showed a high degree of correlation between the two readings. The Kappa statistic was calculated for observing intra-examiner reliability for the cervical stages.

Data were entered and analyzed in SPSS for Windows (version 20.0, SPSS Inc. Chicago). Descriptive statistics were applied for the calculation of mean chronological and dental age. The Shapiro-Wilk test was applied to determine the normality of the data, which showed a non-normal distribution. The Mann-Whitney U test was used to compare the mean chronological and dental age at different cervical stages between the sexes. Spearman’s correlation coefficient was used to assess the correlation between chronological and dental age and cervical stages with Demirjian and Nolla’s stages.

## RESULTS 

ICC showed a high degree of correlation for Demirjian and Nolla’s scores: 0.800 and 0.915, respectively. The dental age, as determined by DI, showed a high degree of correlation (0.868) as it did with NS (0.897). The Kappa statistic showed a substantial agreement (0.788) when the cervical stages were evaluated. 

The mean chronological and dental ages at different cervical stages for males and females are given in Table 1. Chronological age showed a significant difference (*p*= 0.003) between genders at CS5. The dental age as calculated with NS showed a significant difference between the sexes (*p*= 0.007) at CS3.

**Figure 1:**
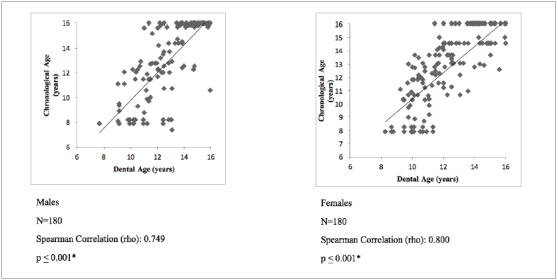
Correlation between Chronological Age and DI.

**Figure 2:**
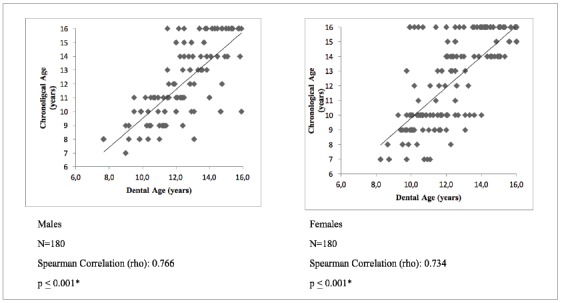
Correlation between Chronological Age and NS.

Spearman’s correlation showed a moderate positive correlation (rho = 0.749) between chronological and dental age according to the DI in males; whereas, a strong positive correlation (rho = 0.800) was seen in females (Fig 1). Spearman’s correlation showed a moderate positive correlation between chronological and dental age according to NS in males (rho = 0.766) and females (rho = 0.734) (Fig 2). 

**Figure 3:**
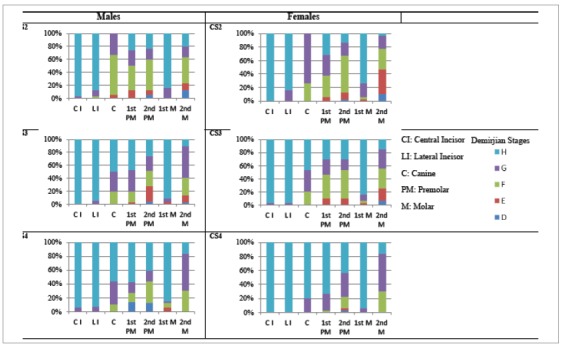
Percentage distribution of Demirjian stages around the peak growth stages.

**Figure 4:**
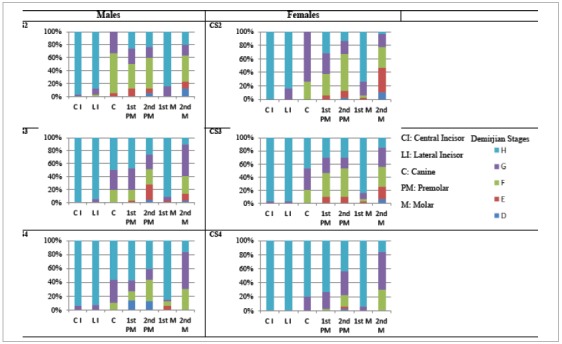
Percentage distribution of Nolla stages around the peak growth stages.

The percentage distribution of the calcification stages of each tooth among the peak growth cervical stages was determined for the Demirjian (Fig 3) and Nolla’s method (Fig 4). According to the DI, the mandibular canine, first premolar and second premolar are calcified by CS3, whereas the second molar is in stage G. NS also showed a similar percentage distribution. 

The Spearman’s correlation of the cervical stages with the Demirjian and Nolla’s stages is given in Table 2, which shows that the mandibular canine has the highest correlation with the DI in males (rho = 0.818) and in females (rho = 0.833). The mandibular second premolar showed the highest correlation with NS in males (rho = 0.654) and in females (rho = 0.664).

## DISCUSSION 

This study was conducted to determine the relationship between the cervical stages and the calcification stages of permanent mandibular teeth. A number of methods have been proposed over the years to determine skeletal maturity, including the hand-wrist radiographs,^20^ the elbow radiograph^21^ and morphology of the frontal sinus.^22^ The hand-wrist radiograph was known to be a reliable technique to determine the growth stages until Lamparski^23^ developed the cervical vertebral maturation (CVM) method. Over time, studies were able to validate the use of the CVM method as it consistently demonstrated good reliability with the hand-wrist radiograph.^24^^-^^26^

Due to the variations in populations and ever changing conditions, it is necessary to correctly determine the growth stage of the individual.^27^ The knowledge of girls maturing earlier than boys has been established for many years.^28^ Females also show an advanced developing dentition.^29^^,^^30^ The comparison of mean chronological age at different cervical stages showed a significant difference at CS5, which indicated that female subjects attained skeletal maturity, on average, one year before male subjects. In contrast to previous findings, we found that the calcification of mandibular teeth occurs earlier, exhibiting stages G and H by the time they have reached CS3. ^31^^,^^32^ Hence, it is more likely to find younger patients with a complete permanent dentition at an earlier chronological age.

The correlation between the cervical and dental maturation stages of individual mandibular teeth has been seen by different authors,^3^^,^^5^^,^^7^^,^^11^ but there is no consensus about which tooth is the most accurate predictor of dental age. Litsas and Loucchese^33^ found the central incisor, lateral incisor and the first molar to be unhelpful in identifying the growth stages of an individual as the root formation and apex closure of these teeth is complete by CS2, which was also observed in our study. 

The DI has been used to correlate the canine, second premolars and second molars with the cervical stages. Goyal et al^12^ investigated the association between the mandibular canine and the cervical maturation stages to help determining the onset of the pubertal growth spurt. The calcification of the canine was identified to represent the onset but not the peak of the adolescent growth spurt, as its root completion occurs by 13 years of age. The canine stages, which could be considered as indicative of growth status in the beginning stages (stages E and F), were found to have a more reliable significance in male than female subjects. A strong correlation was found with the DI and cervical stages for the mandibular canine. Mittal et al^34^ found that with second mandibular premolars, stage F of the DI corresponded to CS3. Kumar et al^13^ evaluated skeletal maturity using the mandibular second molar stages to reveal that Stage E included the highest percentage distribution (68.75%) at stage 2 of the CVMI. Stages F and G were almost equally distributed for CVMI stage 3. In this study, the mandibular canine was in Demirjian stages G and H by the time CS3 was reached. The first and second premolars were found to be in F and G stages. Similarly, the second molar was also found to be in the F and G stages with the Demirjian.

The NS revealed the mandibular canine to be in stages 9 and 10 at CS3 as reported by Sachan et al.^35^ The first and second premolars were found variably in stages 8, 9 and 10. The second molar was found to be in stage 9. The increased number of NS indicates the inaccuracy of this method and this made it difficult to correlate the dental age with cervical stages as noted by other studies.^36^^,^^37^

A strong correlation was found between DI and cervical stages for the mandibular canine, however no such association was noted with the use of NS. The correlation between DI and cervical stages yields a moderately strong association between the first premolar, second premolar and second molar. With the lack of significant association between a single tooth and the DI and NS, the prediction of skeletal maturity using the dental age still seems to harbor ambiguity. It is needless to say these teeth can be used to obtain a close to accurate determination of the skeletal maturity. 

The DI has shown good correlation when used in different populations, but the results have varied and this was recognized quite rapidly after its’ development.^3^^,^^5^ This disparity may exist due to inter-examiner variation that has been noted between different authors. Nevertheless, it is the most commonly utilized index to determine dental age and its’ use has been recommended over other maturity indices.^38^

The DI was compared with the NS to determine which of the two could better identify the stages of growth in our population. The dental age calculated using the DI was found to have a non-significant difference when compared to the cervical stages. A significant difference was found with the NS at CS3. This indicates that NS are more sensitive to the peak of the adolescent growth spurt and better estimates the stages of growth. 

A consistent problem that the DI has suffered from is that it generally overestimates the chronological age of individuals, which was in concordance with our study.^39^^,^^40^ In contrast, the NS were more accurate in the assessment of the chronological age of patients despite previous studies that have reported that it discriminated the dental age between sexes.^41^ A population-based study with a larger sample size could help developing and modifying the existing Demijian tables and NS that are specific to our country. A single center study such as this makes it difficult to generalize the results and requires a multicenter approach.

The identification of the pubertal growth period is important when making clinical decisions, especially for dentofacial orthopedic treatment planning. Myofunctional appliances can bring about greater skeletal response during this period, in contrast to greater dental changes after the growth has ended. Therefore, it is wise to begin treatment during the CS2-3 period. The assessment of the calcification of teeth can be easily incorporated into clinical practice as the dental panoramic radiograph is done for patients presenting for dental treatment.

## CONCLUSION 

This study was conducted to determine an association between the stages of cervical vertebrae maturation and the calcification stages of permanent mandibular teeth, and whether these stages can be used as indicators to determine skeletal maturity.

» The comparison of skeletal age between the genders showed that females reach skeletal maturity on average one year earlier than males.
» The dental ages between the sexes revealed that females are dentally advanced as compared to males.
» The NS were able to identify CS3, which represents the period of maximum growth.
» The DI was able to identify stage F of the mandibular canine to be indicative of CS2 growth stage, and NS identified stage 9-10 to be indicative of CS2 and CS3.

The DI was able to identify stage F and G to be indicative of the CS3 for the first and second premolars and second molars. NS were found to be between stages 8-10 for these teeth.
